# Hospital Meetings, &c.

**Published:** 1898-03-19

**Authors:** 


### HOSPITAL MEETINGS, &C.

### ROYAL ALBERT ORPHAN ASYLUM.

The annual meeting of the Royal Albert Orphan Asylum was held at the offices, 62, King William Street, E.C., on Wednesday, the 9th inst., Colonel the Hon. Charles Eliot in the chair. In presenting the report for the year 1897 the chairman said that the accounts showed a deficit of £1,266, owing in a measure to the numerous Jubilee funds which had been raised during the year. The items which showed the greatest decrease were donations (£2,211 in 1896, and £746 in 1897) and legacies (£1,981 and £440 respectively). Annual subscriptions showed a falling off of nearly £50. The ordinary income amounted to £3,961 in 1896 and to £2,404 in 1897, while the expenditure was £4,091 and £4,110 respectively. The average number of inmates during the year was 180, about the normal number, and the average cost per child worked out at £22 16s. 8d., as compared with £22 9s. 7d. in 1896, and £21 4s. 7d. in 1895. The report and accounts were adopted, and votes of thanks were given to the chairman and the honorary officers. The committee of management was re-elected, and the proceedings shortly afterwards terminated.

### WEST HAM HOSPITAL.

The report for the year 1897, which was adopted at the annual meeting of the West Ham Hospital, held on Wednesday, the 9th inst., states that during the past twelve months 694 in-patients — of whom 576 were surgical and 118 medical cases—were treated, an increase of 114 on the previous year. Of these nearly 60 per cent. came from Stratford and West Ham. In the out-patient department 10,622 cases were admitted by letter, 9,523 were casualties, and 2,268 received dental treatment. As to finance, the year taken in its entirety has been one of reasonable prosperity, the gross expenditure exceeding the income by £168 only. Increases are shown in the receipts from workmen and from entertainments against decreases in the amounts received from congregational collections. The total ordinary income amounted to £4,221, and the ordinary expenditure to £4,196, while £194 was expended on extraordinary repairs and alterations.

